# Thomas Hardy and the tallest – blind -man

**DOI:** 10.1007/s40618-025-02612-6

**Published:** 2025-05-28

**Authors:** Wouter W. de Herder

**Affiliations:** Department of Internal Medicine, Sector of Endocrinology, Rg520 Dr. Molewaterplein 40, Rotterdam, 3015 GD The Netherlands

The English novelist and poet Thomas Hardy (Stinsford, 2 June 1840 – Dorchester, 11 January 1928) published in 1917 in “Satires of circumstances: lyrics and reveries; Moments of vision and miscellaneous verses” the poem “At a Country Fair” [1].

***At a Country Fair***.

*At a bygone Western country fair*.

*I saw a giant led by a dwarf*.

*With a red string like a long thin scarf;*

*How much he was the stronger there*.

*The giant seemed unaware.*

*And then I saw that the giant was blind*,

*And the dwarf a shrewd-eyed little thing;*

*The giant*,* mild*,* timid*,* obeyed the string*.

*As if he had no independent mind*,

*Or will of any kind.*

*Wherever the dwarf decided to go*.

*At his heels the other trotted meekly*,

*(Perhaps—I know not—reproaching weakly)*.

*Like one Fate bade that it must be so*,

*Whether he wished or no.*

*Various sights in various climes*.

*I have seen*,* and more I may see yet*,

*But that sight never shall I forget*,

*And have thought it the sorriest of pantomimes*,

*If once*,* a hundred times!*

The giant / tall man described in the poem was Joseph Neal Sewell who was also known as “the Lincolnshire Gigantic Youth”. He was born as an illegitimate child in the village of Scambellsby, near Horncastle, in the county of Lincoln, England on 18 February 1805. By the age of 14, he weighed 20 stone (127 kg.) and was exhibited at fairs as a “fat boy”. At the age of 24, he was 7 feet 4 inches (223.5 cm.) tall, 2 foot 9 inches (84 cm.) broad and weighed 37 stone (235 kg.). He became blind in 1825 during a visit of Swansea, Wales when he was affected with typhus. After 1825, he earned a living by exposing himself to the British public. Touring with him and guiding him was the short stature performer Natty Farnham who stood at only 37 inches (94 cm.) tall and weighed 68 pounds (31 kg.). Joseph Neal Sewell died because of epileptic seizures in Swansea, Wales, on 5 July 1829, aged only 24 [2]. According to the Welsh newspaper “Monmouthshire Merlin” of 11 July 1829: “On Friday evening he complained of being unwell, and shortly afterwards had several epileptic fits, which so completely shook the caravan, that the men were obliged to secure the wheels to prevent it from falling” [3]. His corpse was transported to Taunton, Somerset, England, where he was buried on July 13, 1829 on the north side of St. Mary Magdalene Church (nowadays Taunton Minster), where his long grave was still to be “seen for many years”. In the Museum of Somerset, Taunton Castle, Taunton, his shoes are on display.

Most likely, Joseph Neal Sewell suffered from acromegaly - gigantism caused by a pituitary tumor. His blindness can be attributed to compression of the optic nerve by the tumor. His seizures might have had several different potential causes: meningitis, hydrocephalus, or tumor infiltration of the brain [2].

The poem was written by Tomas Hardy in the first person, referring to a past event (“bygone”), but still in Hardy’s living memory (“but that sight never shall I forget”). However, it is obvious that this was not his personal experience (Joseph Neal Sewell died before Thomas Hardy was born), but rather recounted to him and written as if he had witnessed the scene himself [2].
